# Brief Review on COVID-19: The 2020 Pandemic Caused by SARS-CoV-2

**DOI:** 10.7759/cureus.7386

**Published:** 2020-03-24

**Authors:** Damian N Valencia

**Affiliations:** 1 Internal Medicine, Kettering Medical Center, Dayton, USA

**Keywords:** covid-19, sars-cov-2, corona virus, 2019-ncov, novel coronavirus, chloroquine, acei, arb, remdesivir, corticosteroids

## Abstract

Severe acute respiratory syndrome coronavirus 2 (SARS-CoV-2) is the virus responsible for the coronavirus disease of 2019 (COVID-19). First identified in Wuhan (Hubei, China) in December of 2019, it has since been declared a pandemic by the World Health Organization in March of 2020. In this study, we will provide a brief review of viral origin, identification, symptoms, transmission, diagnosis, and potential treatment strategies for the newly identified SARS-CoV-2 strain.

## Introduction and background

Severe acute respiratory syndrome coronavirus 2 (SARS-CoV-2) is the virus responsible for the coronavirus disease of 2019 (COVID-19). First identified in Wuhan (Hubei, China) in December of 2019, it has since been declared a pandemic by the World Health Organization (WHO) in March of 2020 [1-2].

**Figure 1:**
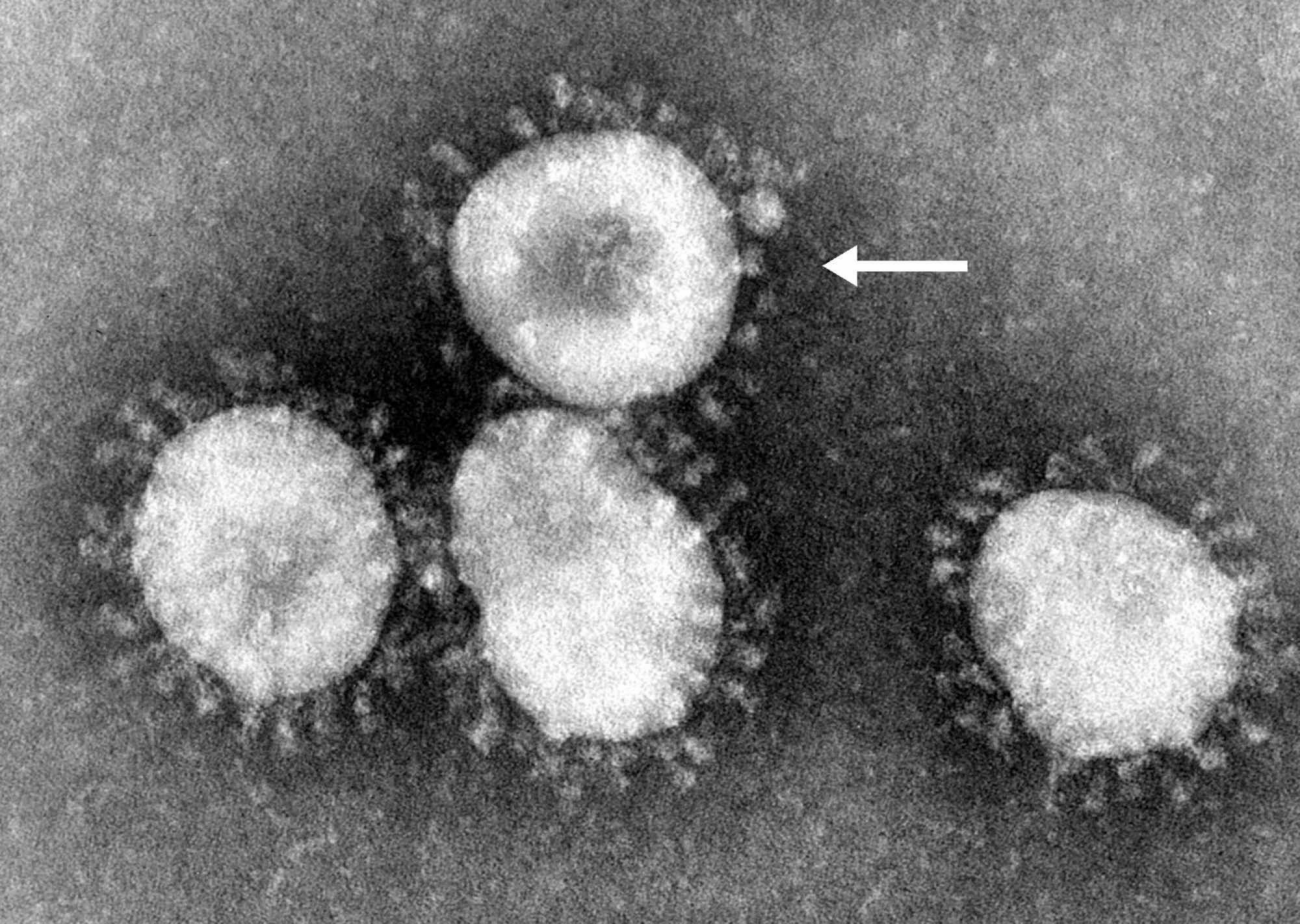
SARS-CoV Electron microscopy image of SARS-CoV, with the arrow pointing at a single virion. Photo credit to Dr. Fred Murphy. This media comes from the Centers for Disease Control and Prevention's (CDC) Public Health Image Library (PHIL), identification number 4814 (https://phil.cdc.gov/Details.aspx?pid=15523). SARS-CoV, severe acute respiratory syndrome coronavirus

First discovered in the 1960s, coronaviruses (Coronaviridae) are a family of enveloped positive-sense single-stranded ribonucleic acid (RNA) viruses [3]. The genome size of this viral group ranges between 27 and 34 kilobases, which is larger than most other RNA viruses [4]. The name Coronavirus originates from the Latin word corona, meaning “crown” or “halo”, due to its characteristic appearance under two-dimensional transmission electron microscopy. Coronaviruses have club-shaped spike peplomers covering their surfaces (Figure 1) [5].

**Figure 2:**
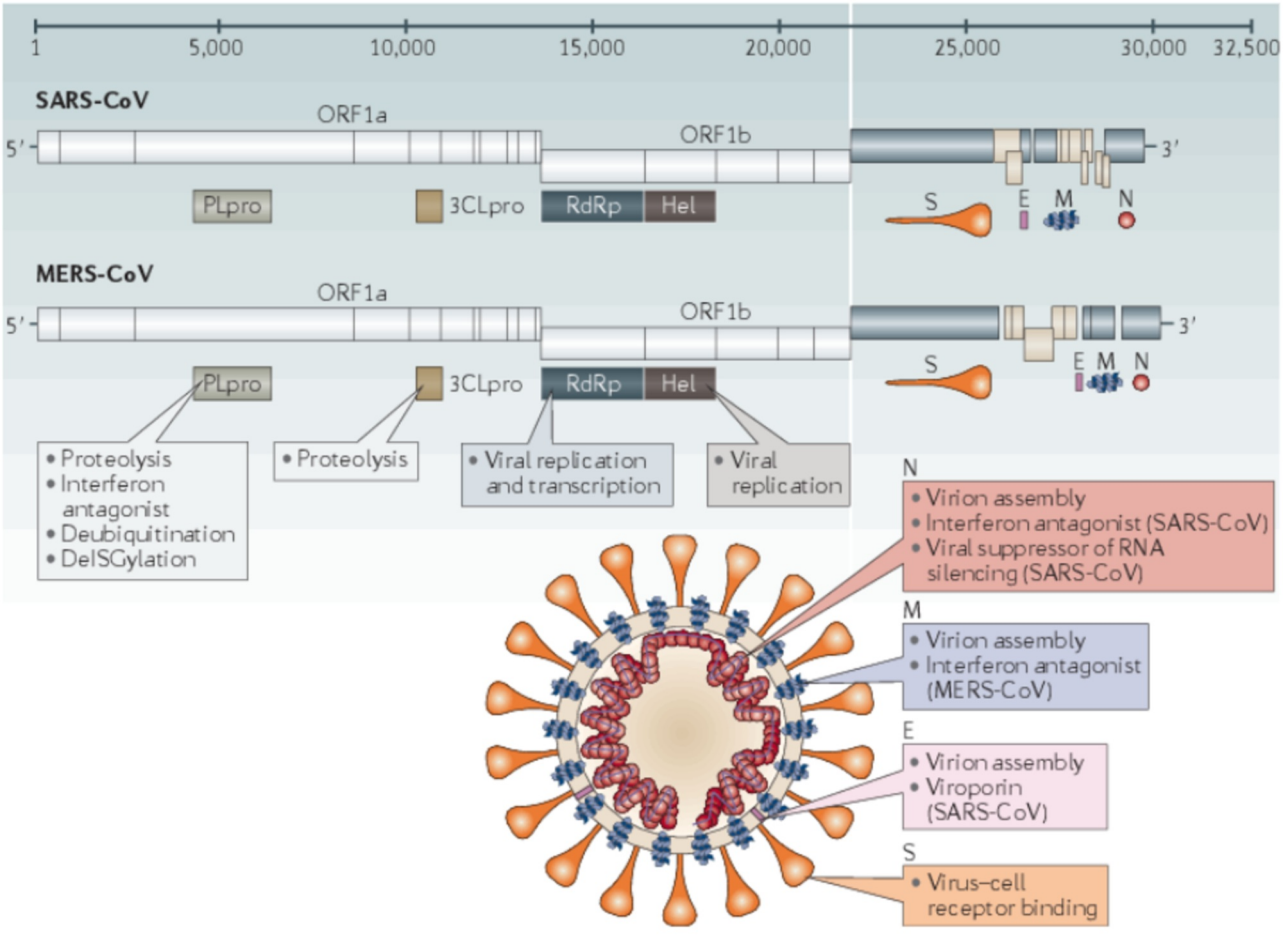
Genomes and structures for SARS-CoV and MERS-CoV The image shows the key SARS-CoV and MERS-CoV virion components, along with their genome sequencing. Photo credit to Zumla et al. [9]. SARS-CoV, severe acute respiratory syndrome coronavirus; MERS-CoV, Middle East respiratory syndrome coronavirus

Since their discovery, seven human pathogenic strains have been identified. Within the Coronaviridae family and Orthocoronavirinae subfamily, the Alphacoronavirus and Betacoronavirus are transmissible to humans. The Alpha- and Betacoronavirus strains are thought to have originated from the bat species (Rousettus leschenaultii) [6-8]. Clinical presentation can vary widely, ranging from mild cold-like symptoms to severe respiratory distress and death. The Alphacoronavirus strains 229E and NL63, along with the Betacoronavirus strains OC43 and HKU1, tend to cause only mild symptoms. The Betacoronavirus strains MERS-CoV (Middle East respiratory syndrome coronavirus), SARS-CoV (severe acute respiratory syndrome coronavirus), and SARS-CoV-2 are known for causing severe respiratory distress. In recent history, several outbreaks have occurred related to these Betacoronavirus strains. Figure 2 depicts the genomes and structures for SARS-CoV and MERS-CoV [9].

Human-to-human transmission primarily occurs through close contact and through respiratory droplets [2]. Similar to many other viral particles, transmission is increased at lower temperatures. Viral-laden droplets are more effectively produced due to increased evaporation at lower relative humidity, allowing for viral particles to remain airborne for longer [10]. Once viral particles enter the respiratory tract, the virus attaches to pulmonary cells followed by endocytosis.

**Figure 3:**
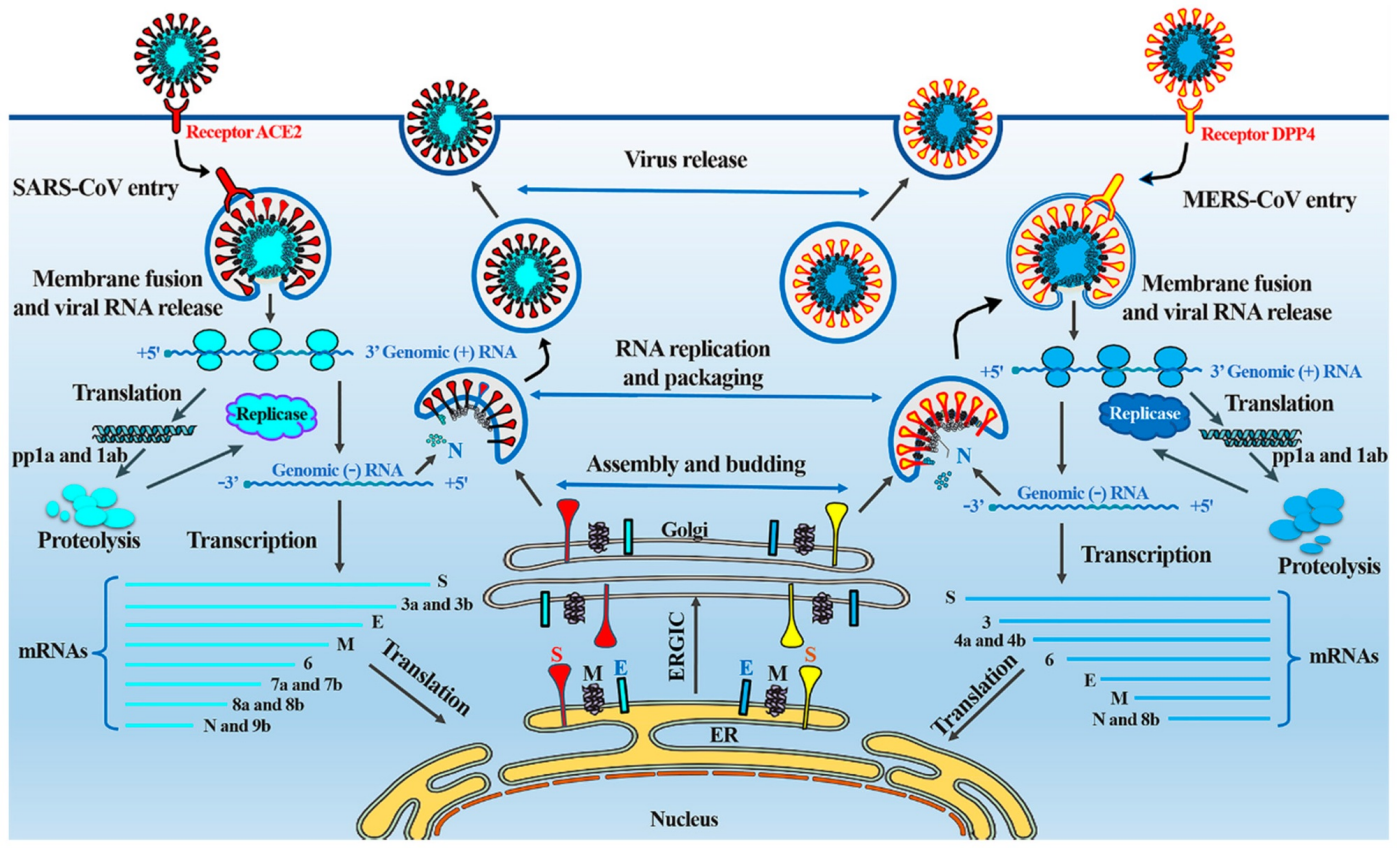
Replication cycle of SARS-CoV and MERS-CoV This image details the replication cycle of SARS-CoV and MERS-CoV. Photo credit to Zumla et al. [12].

Both SARS-CoV and MERS-CoV enter cells through an endocytosis pathway, using surface spike (S) proteins to bind to the angiotensin-converting enzyme 2 (ACE-2) and dipeptidyl peptidase 4 (DPP4) receptors on the ciliated bronchial epithelial cells and type II pneumocytes, respectively [11]. Once the virus enters the host cell, the viral RNA is exposed. Open reading frames 1a and 1ab (ORF1a and ORF1ab) are translated, producing polyproteins (pp1a and pp1ab). These polyproteins are later cleaved to form structural proteins for the RNA replicase-transcriptase complex, which is responsible for the replication and transcription of viral RNA. Viral nucleocapsids are assembled and bud from the lumen of the endoplasmic reticulum Golgi intermediate compartment (ERGIC). As viral nucleocapsids encase viral RNA to produce new coronavirus virions, they are exocytosed, completing the replication cycle. Viral replication is summarized in Figure 3 [11-13].

## Review

Here we will present a brief review of viral origin, identification, symptoms, transmission, diagnosis, and potential treatment strategies for the newly identified SARS-CoV-2 strain.

Origin

In December of 2019, a cluster of atypical pneumonia cases were reported in Wuhan, China, with the first known case recorded on December 1 [14]. The majority of patients diagnosed with this atypical pneumonia had links to the Huanan Seafood Market, suggesting a zoonotic origin [15-17]. Some reports indicate early rapid spread, with cases doubling every 7.5 days [18]. On January 30, 2020, the WHO declared a public health emergency of international concern as cases began to spread around the world [1]. On March 11, 2020, the WHO declared the outbreak of SARS-CoV-2 a pandemic [1].

Identification

**Figure 4:**
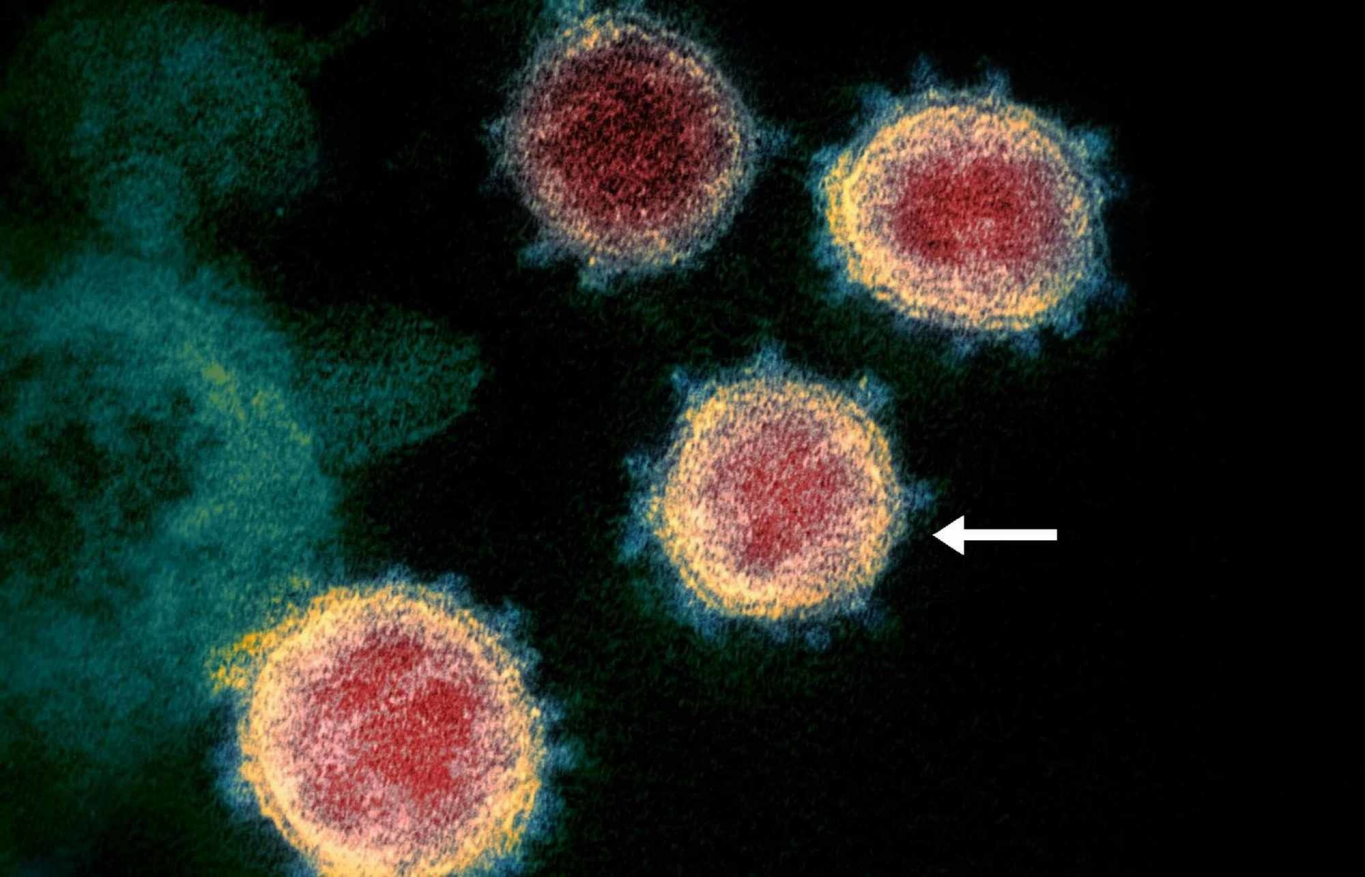
Electron microscopy image of SARS-CoV-2 virions Electron microscopy image of SARS-CoV-2, with the arrow pointing at a single virion. Photo credit to the National Institute of Allergy and Infectious Diseases (NIAID) Rocky Mountain Laboratories (RML), United States National Institutes of Health (NIH). SARS-CoV, severe acute respiratory syndrome coronavirus

Shortly after investigations began, it was determined that a Betacoronavirus was responsible, which was identified as SARS-CoV-2 (Figure 4).

Prior to its identification, the virus was called the 2019 novel coronavirus (2019-nCoV). Some are suggesting a change of name to human coronavirus 2019 (HCoV-19) to avoid confusion with the recent strain SARS-CoV from 2002. Here, we will refer to the new strain as SARS-CoV-2, as accepted by the WHO and the Centers for Disease Control and Prevention (CDC) [1-2]. This newly identified human strain is thought to be related to the bat and pangolin coronavirus as well as SARS-CoV [19-22]. Genetic analysis has placed the virus in the genus Betacoronavirus and subgenus Sarbecovirus (lineage B), which confirms its likely origin to the bat coronavirus (BatCoV RaTG13) [22]. Further analysis has revealed only one amino acid difference between SARS-CoV and the pangolin Coronavirus (Pangolin-CoV), suggesting a possible intermediate host [23].

Symptoms

Patients who test positive for SARS-CoV-2 and are symptomatic are diagnosed with COVID-19. Symptoms can vary drastically; they include fever (99%), chills, dry cough (59%), sputum production (27%), fatigue (70%), lethargy, arthralgias, myalgias (35%), headache, dyspnea (31%), nausea, vomiting, anorexia (40%), and diarrhea [22,24]. Some carriers may be asymptomatic, whereas others can experience acute respiratory distress syndrome (ARDS) and death [22,24]. Severity seems to also vary with age, disproportionately affecting those of advanced age and those with pre-existing chronic medical conditions (Table 1) [25].

**Table 1:** Hospitalization, ICU admission, and case fatality percentages for reported COVID–19 cases by age group. These data comes from the Centers for Disease Control and Prevention, the Morbidity and Mortality Weekly Report (MMWR) dated February 12 to March 16, 2020, as service marks of the U.S. Department of Health and Human Services [25]. *The lower bound of range is the number of persons hospitalized, admitted to ICU, or who died among total in the age group; the upper bound of range is the number of persons hospitalized, admitted to ICU, or who died among total in the age group with known hospitalization status, ICU admission status, or death. ICU, intensive care unit; COVID-19, coronavirus disease of 2019

Age group (years) (no. of cases)	%*		
	Hospitalization	ICU admission	Case fatality
0–19 (123)	1.6–2.5	0	0
20–44 (705)	14.3–20.8	2.0–4.2	0.1–0.2
45–54 (429)	21.2–28.3	5.4–10.4	0.5–0.8
55–64 (429)	20.5–30.1	4.7–11.2	1.4–2.6
65–74 (409)	28.6–43.5	8.1–18.8	2.7–4.9
75–84 (210)	30.5–58.7	10.5–31.0	4.3–10.5
≥85 (144)	31.3–70.3	6.3–29.0	10.4–27.3
Total (2,449)	20.7–31.4	4.9–11.5	1.8–3.4

Transmission

Transmission occurs primarily through respiratory droplets, but it can also occur through contact with contaminated surfaces [2]. Viable viral particles may remain on stainless steel and plastics for up to 72 hours after application [26]. Currently, the CDC recommends airborne and droplet precautions for all healthcare providers who come in contact with potential COVID-19 patients [2]. Several public measures have been taken at the local and federal government level in the United States to reduce the rates of transmission, including social distancing and self-isolation.

Incubation periods may vary but have been known to be between 1 and 14 days for other coronaviruses. To date, the median observed incubation period for SARS-CoV-2 appears to be 5.1 days (95% confidence interval [CI]: 4.5-5.8 days), with 97.5% of those who develop symptoms doing so within 11.5 days (95% CI: 8.2-15.6 days) of infection [27]. Although the risk of transmission from an asymptomatic individual may be low, it is still possible. The basic reproduction number (R0), or the number of cases directly generated by one case in a population where all individuals are susceptible, has been reported to be between 2.13 and 4.82, which is similar to SARS-CoV [28]. At the cellular level, once viral particles enter the respiratory tract, like SARS-CoV, SARS-CoV-2 uses the ACE-2 receptors for pulmonary cell entry [29]. ACE-2 is a type 1 transmembrane metallocarboxypeptidase, which, under normal physiological circumstances, functions in the degradation of angiotensin II to modulate the renin-angiotensin System (RAS) [30]. The viral S protein binds to the ACE-2 receptor, prompting cellular membrane fusion and endocytosis. This process is dependent on S protein priming by a serine protease (TMPRSS2) in many coronavirus models, potentially identifying a future treatment modality [31-32].

Diagnosis

**Figure 5:**
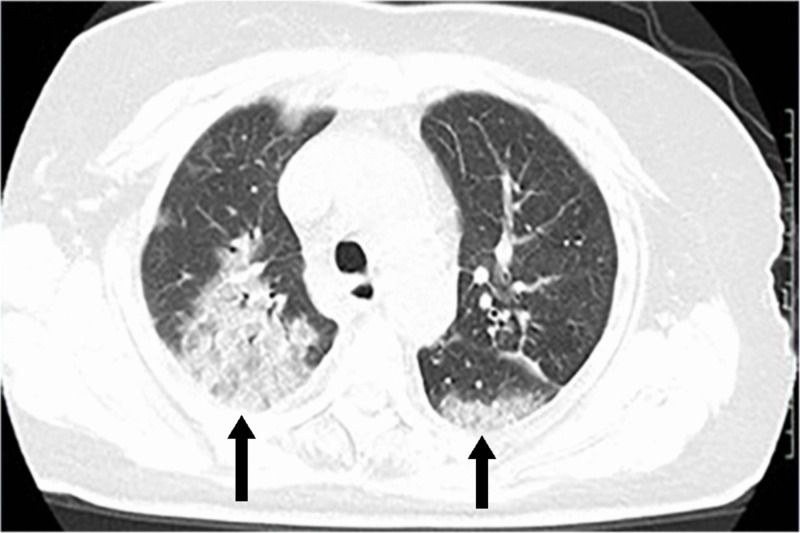
CT of the chest in a COVID-19 patient Axial CT of the chest showing GGO and bilateral posterior opacities with a paving pattern. Photo courtesy of Salehi et al [35]. CT, computed tomography; COVID-19, coronavirus disease of 2019; GGO, ground-glass opacities

Diagnosis is ultimately confirmed by real-time reverse transcription polymerase chain reaction (rRT-PCR) on respiratory or blood samples [33]. Note that rRT-PCR positive-to-negative conversion has been reported at 6.9 ± 2.3 days [33]. Some reports detail imaging findings suggestive of COVID-19, although these findings can be nonspecific and reliability has not yet been established [33-34]. Computed tomography (CT) findings include bilateral multilobar ground-glass opacities, with peripheral posterior distribution, mainly in the lower lung lobes [35]. Less commonly, septal thickening, bronchiectasis, pleural thickening, and subpleural involvement have been reported. As disease progression occurs, repeat CT scan may show multifocal consolidations with a paving pattern (Figure 5) [36].

Treatment

There are currently no definitive therapies or vaccines for the SARS-CoV-2 virus. Management is supportive and, in severe cases, aimed at improving ARDS, which we will not discuss here. Trials are currently underway to identify therapeutic options.

Remdesivir is a nucleotide analog inhibitor of RNA-dependent RNA polymerases, which has previously been shown to have antiviral activity against MERS-CoV and SARS-CoV [36-38]. Studies are currently available that show inhibition of viral replication of SARS-CoV-2 in vitro [36].

Chloroquine, typically used in the context of malarial or autoimmune disease, has also shown promising results. Chloroquine affects glycosylation of the ACE-2 pulmonary cell receptors, impairing viral cell entry [36,39]. Medication-induced pH changes within pulmonary cells (alkalinization) also delays viral replication, as key steps in endosome function are impaired [39]. Similarly, hydroxychloroquine is another less toxic and potentially effective therapy [40]. Trials are currently underway to further evaluate the effectiveness of chloroquine and hydroxychloroquine.

Camostat mesylate, a serine protease inhibitor, has been identified by some as a potential treatment option. Camostat mesylate partially blocks SARS-CoV-2 entry into the pulmonary cells by inhibiting S protein priming and endocytosis [29]. Follow-up studies on treatment with camostat mesylate are currently pending.

Tocilizumab is a humanized monoclonal antibody against interleukin-6 receptor (IL-6R Ab), commonly used as an immunosuppressive in the treatment of rheumatoid arthritis and systemic juvenile idiopathic arthritis. It is currently postulated that patients with severe manifestations of COVID-19 experience some degree of cytokine storm, which results in ARDS and death [38,41]. Small studies in China have found some success with the treatment of severe cases of COVID-19 with tocilizumab. The small study found decreased fever, oxygen requirements, and C-reactive protein (CRP), along with improved CT findings [42]. Medication dosing was not provided.

Lopinavir and ritonavir, protease inhibitors, are commonly used in the treatment and prevention of human immunodeficiency virus (HIV) and acquired immunodeficiency syndrome (AIDS). Randomized, controlled, open-label trials on confirmed positive COVID-19 adult patients with ARDS have been performed using a 14-day course of lopinavir and ritonavir 400-100mg twice daily. No benefit has been observed beyond the standard of care [43]. Some postulate that the combination of lopinavir and ritonavir may become more effective with the addition of interferon-beta (INFb) [38]. Further studies are required to confirm this finding.

Nitazoxanide is a broad-spectrum antiparasitic and antiviral agent used in the treatment of various helminthic, protozoal, and viral infections. Nitazoxanide was found to inhibit SARS-CoV-2 at low micromolar concentrations in vitro [36]. Further studies are required to prove in vivo efficacy.

Medication advisory

Caution should be used when using corticosteroids in COVID-19 patients. Previous data suggest decreased viral clearance of both MERS-CoV and SARS-CoV, potentially prolonging the course of illness [44-45]. No mortality benefit has been appreciated in non-ARDS COVID-19 patients [46].

There has been some speculation regarding non-steroidal anti-inflammatories (NSAIDs), specifically ibuprofen, causing up-regulation of ACE-2 receptors, although no studies are available at this time to suggest an increased risk of SARS-CoV-2 [47]. Similarly, groups have voiced concern over ACE inhibitor (ACEi) and angiotensin receptor blocker (ARB) therapy. This concern is due to their mechanism of action and up-regulation of the ACE-2 receptor, which is used by SARS-CoV-2 in cell entry [47]. No studies have been performed to evaluate this theoretical risk. Currently, the expert opinion recommendation for patients on ACEi or ARB therapy is to continue their current drug regimen. Many societies have made statements regarding this matter and are detailed in Table 2 [48].

**Table 2:** Society recommendations List of all current professional society recommendations regarding ACEi and ARB therapy in the context of COVID-19 [48]. ACEi, angiotensin-converting enzyme inhibitor; ARB, angiotensin receptor blocker; COVID-19, coronavirus disease of 2019

Society	Summary of Recommendations	Statement Date
European Society of Hypertension	Recommend continuing ACEi/ARB due to lack of evidence to support differential use in COVID-19 patients. In those with severe symptoms or sepsis, antihypertensive decisions should be made on a case-by-case basis taking into account current guidelines.	March 12, 2020
European Society of Cardiology Council on Hypertension	Strongly encourage continuing ACEi/ARB due to lack of evidence to support discontinuing.	March 13, 2020
Hypertension Canada	Recommend continuing ACEi/ARB due to lack of evidence that patients with hypertension or those treated with ACEi/ARB are at a higher risk of adverse outcomes from COVID-19 infection.	March 13, 2020
Canadian Cardiovascular Society	Strongly encourage continuing ACEi/ARB and angiotensin receptor neprilysin inhibitors due to lack of clinical evidence to support withdrawal of these agents.	March 15, 2020
The Renal Association, United Kingdom	Strongly encourage continuing ACEi/ARB due to unconvincing evidence that these medications increase risk.	March 15, 2020
International Society of Hypertension	Strongly recommend that the routine use of ACEi/ARB to treat hypertension should not be influenced by concerns about COVID-19 in the absence of compelling data that ACEi/ARB either improve or worsen susceptibility to COVID-19 infection, nor do they affect the outcomes of those infected.	March 16, 2020
American College of Physicians	Encourage continuing ACEi/ARB because there is no evidence linking them to COVID-19 disease severity, and discontinuation of antihypertensive therapy without medical indication could in some circumstances result in harm.	March 16, 2020
Spanish Society of Hypertension	Recommend that ACEi/ARB should not be empirically stopped in patients who are already taking them; in seriously ill patients, changes should be made on a case-by-case basis.	March 16, 2020
American Heart Association	Recommend continuing ACEi/ARB for all patients already prescribed them.	March 17, 2020
Heart Failure Society of America	Recommend continuing ACEi/ARB for all patients already prescribed them.	March 17, 2020
American College of Cardiology	Recommend continuing ACEi/ARB for all patients already prescribed them.	March 17, 2020
European Renal Association	Recommend continuing ACEi/ARB in COVID-19 patients due to lack of evidence to support differential use and the discontinuation of ACEi/ARB in COVID-19 patients.	March 17, 2020
European Dialysis and Transplant Association	Recommend continuing ACEi/ARB in COVID-19 patients due to lack of evidence to support differential use and the discontinuation of ACEi/ARB in COVID-19 patients.	March 17, 2020
American Society of Pediatric Nephrology	Strongly recommend continuing ACEi/ARB until new evidence to the contrary becomes available.	March 17, 2020
High Blood Pressure Research Council of Australia	Recommend continuing routine use of ACEi/ARB. Patients should not cease blood pressure lowering medications unless advised to do so by their physician.	March 18, 2020

Outcomes

Case fatality varies geographically, and final mortality estimates vary weekly as many cases are currently ongoing. Recent data suggest a case fatality between 0.25% and 3.0% [49-50]. Slightly increased rates have been documented in China (3.5%) [49]. Case fatality also varies by age: 14.8% in patients aged ≥80 years, 8.0% in patients aged 70-79 years, and 49.0% in critical cases [50]. It is uncertain whether these figures can predict disease case fatality in the United States, as progression throughout the United States is currently ongoing.

## Conclusions

SARS-CoV-2 is the coronavirus responsible for the COVID-19 pandemic of 2020. It is one of seven human transmissible coronaviruses and is thought to have originated from the bat Coronavirus. The first human cases were documented in Wuhan, China, in December of 2019 and are thought to be a result of transmission through an intermediate host, likely the pangolin. Human-to-human disease transmission primarily occurs through respiratory droplets. Once in the respiratory tract, SARS-CoV-2 enters the pulmonary cells through endocytosis via the ACE-2 receptor. The mean incubation time is 5.1 days (95% CI: 4.5-5.8 days), with 97.5% of those who develop symptoms doing so within 11.5 days (95% CI: 8.2-15.6 days). Symptoms may vary from mild to severe but are typical of other viral illnesses including Influenza. The basic reproduction number is reported to be between 2.13 and 4.82. Those most affected by COVID-19 are those of advanced age and those with pre-existing chronic medical conditions. Final mortality rates are currently unknown, as a large portion of cases have not yet resolved, but estimated case fatality is between 0.25% and 3.0%. Treatment options are limited to supportive care and management of ARDS in severe cases. Ongoing studies are evaluating the efficacy of remdesivir, chloroquine, hydroxychloroquine, camostat mesylate, and tocilizumab as potential therapies. Lopinavir and ritonavir do not appear to be effective. Currently, no vaccine is available, although efforts are in progress to developing a vaccine over the coming year. Caution should be used when using corticosteroids in non-ARDS COVID-19 patients, as no mortality benefit has been observed and viral clearance can be prolonged. The use of ACEis and ARBs should not be discontinued in efforts to prevent or reduce the transmission of SARS-CoV-2 per current society statement.
